# Pushing the Limits: Machine Preservation of the Liver as a Tool to Recondition High-Risk Grafts

**DOI:** 10.1007/s40472-018-0188-7

**Published:** 2018-03-20

**Authors:** Yuri L. Boteon, Simon C. Afford, Hynek Mergental

**Affiliations:** 1 Liver Unit, Queen Elizabeth Hospital Birmingham, University Hospitals Birmingham NHS Foundation Trust, Birmingham, B15 2TH UK; 2 National Institute for Health Research, Birmingham Liver Biomedical Research Centre, Institute of Immunology and Immunotherapy, College of Medical and Dental Sciences, University of Birmingham, Birmingham, UK

**Keywords:** Liver preservation, Organ utilisation, Extended criteria donor, Therapeutic intervention, Functional assessment

## Abstract

**Purpose of the Review:**

Machine perfusion (MP) is a novel technology recently introduced in liver transplantation, redefining the current practice of organ preservation and pushing the limits of high-risk liver utilisation. This review highlights the key benefits of machine perfusion over conventional static cold storage (SCS), demonstrated in human liver research and clinical transplants.

**Recent Findings:**

The first clinical trials have demonstrated both safety and feasibility of MP. The most recent transplant series and result from a randomised trial suggest the technology is superior to SCS. The key benefits include extended period of organ preservation, decreased incidence of early allograft dysfunction and reduction of biliary complications. Normothermic liver perfusion allows viability testing to guide transplantability of the highest-risk organs. This technology also provides opportunities for therapeutic interventions to improve liver function and quality in organs that are currently declined for clinical use.

**Summary:**

Machine perfusion is likely to transform the liver preservation pathway and to improve utilisation of high-risk grafts.

## Introduction

A recent report from the British National Health Service identified that 13% of the 1769 patients registered for liver transplantation in the United Kingdom between 2016 and 2017 died or were removed from the waiting list after becoming too ill for transplantation [1]. Similar data are reported from many other countries reflecting that organ shortage in the context of rising incidence of liver disease is a worldwide phenomenon [2]. To address shortfalls in supply of standard criteria donors, transplant teams have progressively extended the criteria to accept marginal, high-risk organs [3]. Such extended criteria donors (ECD) include donors after circulatory death (DCD), fatty livers, and organs from donors with higher risk behaviour or advanced age with multiple comorbidities [3]. ECD grafts are more susceptible to ischaemic injury and detrimental effects caused by static cold storage (SCS) and are associated with an increased rate of early allograft dysfunction, post-transplant biliary complications and recipient mortality [4–6]. Consequently, an increasing number of procured livers are being declined for transplantation [7]. Steatosis is the leading cause worldwide of livers being discarded (40%), followed by prolonged donor warm ischaemia, poor organ flushing and logistical reasons [8, 9]. Confronting the ongoing decline of standard criteria donors, the increased need for transplantation can be achieve only by more successful transplants from marginal grafts and increased utilisation of high-risk livers [10].

The inferior outcomes of ECD grafts have exposed shortcomings of SCS and directed research towards finding superior means of liver preservation. Following encouraging data from pre-clinical experiments and breakthroughs achieved in heart, lungs and kidney preservation, several teams around the world have reported successful transplantation of machine-perfused livers, and this promising technology has attracted the attention of the transplant community worldwide [3, 11•, 12–14].

Machine liver perfusion has become a rapidly progressing field. Whilst the initial case series demonstrated the technology is safe and feasible in standard criteria livers, subsequently conducted randomised trials have evaluated its efficacy on a whole spectrum of currently utilised organs [11•, 15•, 16, 17•, 18, 19•] Table 1. In keeping with promising experimental data, the current evidence suggests that machine perfusion (MP) will be most beneficial when applied to extended criteria livers.

Recently several teams have started programmes to recondition marginal grafts and to explore frontiers of high-risk organ utilisation. The predominantly used perfusion temperatures are hypothermic (8–12 °C) and normothermic (37 °C), although sub-normothermic and rewarming alternatives have been reported [20–22, 23•].

The hypothermic perfusate is similar to conventional cold storage preservation fluid, and the perfusion technique simpler and cheaper than normothermic machine perfusion. It does not pose additional risks of graft loss compared to SCS and can reveal pre-existing organ damage; however, in a clinical setting, it does not generate the data necessary to assess liver function and transplantability [24, 25].

Sub-normothermic perfusion encompasses the temperature range 12–35 °C, although the majority of experiments were performed at temperatures close to 21 °C [21, 26, 27]. Whilst this perfusion modality delivered promising data in pre-clinical testing, to date, there have not been published data from clinical use. Several teams have investigated graft rewarming, gradually increasing the perfusion temperature from 4 to 21 °C with promising results [22, 28•, 29]. Both these perfusion approaches achieve a partial recovery of liver metabolic function with oxygen requirements that can be met without a dedicated oxygen carrier in the perfusion fluid.

Normothermic machine perfusion of the liver (NMP-L) requires a blood-based perfusate and preserves the liver in near-physiological, fully functioning condition, generating real-time data allowing assessment of its viability [15•, 30, 31]. It can be used as an alternative to SCS and such application has been currently tested in prospective clinical trials across Europe and North America. NMP-L has arguably the most potential to minimise any deleterious effects of the cold ischaemia, but is less flexible, costlier and logistically more demanding [32]. Liver reconditioning provides a more flexible alternative, commencing the perfusion on its arrival at the transplant centre. Several groups have used this technique to resuscitate graft function prior to transplantation, including our team’s work on viability assessment of declined livers [15•, 16]. It can be applied selectively to grafts with suboptimal macroscopic appearance or livers deemed too high-risk for any other reason.

## Extending the Liver Preservation Times

The progressive detrimental effect of cold ischaemia on graft quality is the fundamental limitation of static cold storage. Shortening cold ischaemic times (CIT) to the bare minimum has become a key aspect of successful utilisation of extended criteria organs and many more livers could have been used had the organ arrived to the transplanting centre earlier [33]. The benefit of machine perfusion on removing the constraints due to CIT has previously been shown in the first-in-man NMP-L series where the longest preservation time was close to 19 h [11•].

Subsequently, the first randomised trial comparing NMP-L with SCS performed by the Consortium for Organ Preservation in Europe (COPE WP2 trial, ISRCTN 39731134) identified that clinicians started to exploit this logistic advantage, and livers in the NMP-L group were preserved for significantly longer compared to SCS (11 h 39 min vs 7 h 21 min, *p* < 0.01). Despite this, the organs suffered from less early allograft dysfunction (12.6 vs 29.9%; *p* = 0.002; Nasralla et al., data presented at the British Transplant Society Congress in 2017). Recently, Watson et al reported successful implantation of an extended criteria donor liver 26 h after procurement; in this example, NMP-L was used to assess the organ and the transplant was deferred until extra-hepatic malignancy in the recipient was excluded [34•]. This case report highlights the logistical advantages of NMP-L and the differences in specification of available devices in terms of the maximum liver preservation times [11•, 15•, 30, 35]. Liberation from the CIT constraints might redefine multiple aspects of current liver transplant practice in the future, including new opportunities for supra-regional graft sharing and super-urgent allocation, transformation of donor—recipient matching or streamlining operating theatre logistics and transplant teams’ workforce management.

The limits of extending the NMP-L preservation are yet to be defined, though experimental work has demonstrated successful perfusions beyond 24 h [34•, 35–37]. Whilst 24 h preservation would be sufficient to achieve significant improvements in organ sharing and transplant logistics, machine perfusion may preserve livers for significantly longer. Several research groups have reported experiments with 72-h canine and porcine liver perfusions [36, 38]. The prolonged perfusion will undoubtedly impose new challenges to overcome. For example, red blood cells (RBCs) have clear biophysical limitations when exposed to sheer stress from the circuit tubing and abrasive mechanical insult from centrifugal or roller pumps, leading to an unavoidable degree of haemolysis [39]. To address the problem, our team investigated the feasibility of replacing RBCs with an acellular haemoglobin-based oxygen carrier (HBOC), Hemopure, in a human model of NMP-L. Our results demonstrated similar metabolic and flow parameters, whilst the HBOC-perfused livers extracted more oxygen than those perfused with RBCs (O_2_ER 13.75 vs 9.43% × 10^5^ per gram of tissue, *p* = 0.001) without increased apoptosis or necrosis, tested in vitro hepatic cell lines [32]. Exploring strategies to maintain prolonged perfusion will clearly require intensive research to optimise the perfusate composition and device design, but achieving this goal will open new possibilities for using machine perfusion as a tool for therapeutic intervention and regeneration of the highest-risk liver grafts.

## Pushing the Limits of High-Risk Organ Utilisation

While the initial series of machine perfused transplants included essentially standard criteria organs, the technology’s greatest benefit is preservation of marginal livers [40]. Guarrera et al. applied hypothermic perfusion to 31 extended criteria livers donated after brainstem death (DBD) and, despite their model not providing the organs with oxygen, the authors observed a decreased rate of biliary complications and hospital stay compared to matched SCS controls [19•]. Compelling data were reported by the Zurich group using hypothermic oxygenated perfusions (HOPE) of DBD livers [41]. Consequently, Dutkowski and colleagues achieved nation-wide adoption of HOPE by the Swiss healthcare for all DCD livers, utilising these marginal organs with superior outcomes. Comparison of their results with matched controls from the Netherlands and UK demonstrated the incidence of non-anastomotic biliary strictures in 25 HOPE-perfused livers compared to 50 SCS matched DCD transplants was significantly lower (0 vs 22%, *P* = 0.015), together with superior 1-year graft survival (90 vs 69%, *P* = 0.035) [17•]. Most recently, the data from the COPE WP2 multi-centre European study enrolling 272 donors (consisting of 194 DBD and 78 DCD organs) showed significantly lower organ discard rate in the normothermic arm (NMP-L 16 vs SCS 32 livers; *p* = 0.01; Nasralla et al., data presented at the British Transplant Society Congress in 2017). The data regarding biliary complications and survival at 6-months from this trial are not yet available Table 1.

**Table 1:** Evidence and benefits of machine perfusion over the static cold storage

Study endpoint*	Machine perfusion technique				
	Hypothermic non-ogygenated perfusion	Hypothermic oxygenated perfusion^@^	Normothermic preservation	Normothermic reconditioning	Sub-normothermic reconditioning
Safety and feasibility: standard criteria donors	PCS, *n*^§^ = 20 [ref: 33]	PCS, *n* = 8 [ref: 34•]	RCT^&^, *n* = 220 [Nasralla et al, data submitted; ref: 11•, 14]	PCS, *n* = 12 [ref: 15•, 35]	PCS, *n* = 6 [ref: 42]
Safety and feasibility: extended criteria donors	PCS, *n* = 31 [ref: 19•]	PCS, *n* = 25 [ref: 17•, 34•, 38]	RCT^&^, *n* = 220 [Nasralla et al, data submitted; ref: 11•, 14]	PCS, *n* = 12 [ref: 15•, 35]	PCS, *n* = 6 [ref: 42]
Efficacy: early graft dysfunction	PCS, *n* = 31 [ref: 19•, 33]	PCS, *n* = 25 [ref: 17•, 34•, 38]	RCT^&^, *n* = 220 (Nasralla et al, data submitted)	PCS, *n* = 12 [ref: 15•]	PCS, *n* = 6 [ref: 42]
Efficacy: non-anastomotic biliary strictures	No data available yet	PCS, *n* = 25 [ref: 17•, 34•]	No data available yet	PCS, *n* = 12 [ref: 35]	No data available yet
Extending the limits: preservation time	Not applicable	No data available yet	PCS, *n* = 20 [ref: 11•]	PCS, n = 12 [ref: 15•]	No data available yet
Graft functional assessment	Not possible	Not possible (no real-time assessment available)	RCT^&^, *n* = 220 (Nasralla et al, data submitted)	PCS, *n* = 12 [ref: 15•, 35]	No data available yet
High-risk liver utilisation	PCS, *n* = 31 [ref: 19•, 33]	PCS, *n* = 25 [ref: 17•, 34•, 38]	No data available yet	PCS, *n* = 12 [ref: 15•, 35]	PCS, *n* = 6 [ref: 42]
Therapeutic interventions	No data available yet	No data available yet	No data available yet	No data available yet	No data available yet

*PCS*, prospective cohort study; *RCT*, randomised controlled trial; *DBD*, donation after brain death; *DCD*, donation after circulatory death

*The table includes the key evidence published to date in each particular perfusion technique and the static cold storage used as a reference for comparison

^§^*n* number relates to the largest published series

^&^COPE WP2 trial (ISRCTN 39731134)

^@^Multi-centres European randomised controlled trials in progress (HOPE, NCT01317342 and D-HOPE, NCT02584283)

The development of normothermic perfusion enabled objective assessment of the liver function, advancing the graft selection process, and several teams researched the development of criteria for viability assessment including perfusate pH, bile production, transaminase levels or lactate clearance.

Through pre-clinical experiments, our team observed a close relationship between liver function and perfusate lactate metabolism. The organs able to achieve lactate clearance below 2.5 mmol/L within 120 min of commencing perfusion retained physiological perfusate acid base milieu and did not require any interventions to maintain extended perfusion. Transforming the observations into clinical practice, we proposed composite criteria, based on the lactate clearance and bile production in combination with vascular flows and macroscopic appearance [16]. These were applied on a pilot series of six livers declined for transplantation by all the UK transplant centres and subjected to normothermic perfusion. The study enrolled four DCD and two DBD grafts, commencing perfusion after a period of CIT ranging from 387 to 474 min [15•]. The liver viability was assessed within a 2-h window, and five of the organs met the criteria and were successfully transplanted. In all recipients, we observed immediate graft function recovery with a short hospital stay, and normalised liver function tests within the first post-transplant month [15•]. We have consequently conducted the VITTAL clinical trial (VIability Testing and TrAnsplantation of discarded donor Livers; NCT02740608), further testing the boundaries of the highest-risk organ utilisation by criteria principally based on lactate metabolism, extending the assessment period up to 4 h.

NMP-L provides the opportunity to explore multiple parameters relevant to liver function, and it is still to be determined which can best predict post-transplant outcomes. The Cambridge group advocated graft assessment based on the perfusate transaminases and bile pH [42]. The authors observed a significant correlation between the alanine transaminase (ALT) in the perfusate measured after 2 h perfusion and the peak ALT post-transplant levels within the first week [42]. In this report, Watson et al. also hypothesise that the liver capacity to produce an alkaline bile (pH > 7.4) might be a marker of good cholangiocyte function, possibly allowing selection of organs with a low risk of developing intrahepatic cholangiopathy. If validated, this observation might revolutionise DCD liver utilisation, preventing futile transplantation of grafts with a limited life span. We expect that future organ functional assessment will include more sophisticated methods and markers based on perfusate omics or microRNA analyses [43, 44].

Although the viability testing data have already shown benefits of NMP-L in high-risk graft utilisation and reduced risk of early graft failure, the evidence that MP improves long-term transplant outcomes is still elusive. A pertinent cohort in which to study this subject are DCD livers. To date, the only clinical evidence comes from retrospective observations published by the Zurich and Groningen groups [17•, 41, 45•].

The experimental evidence from normothermic perfusion suggests a protective effect of peri-biliary glands [46–48]. The evaluation of the biliary complication data from the randomised COPE trials is eagerly awaited.

## New Frontiers and Therapeutic Interventions during Machine Perfusion

Designing a machine perfusion clinical trial powered to demonstrate differences in post-transplant graft or patient survival is very challenging, and researchers often use a validated surrogate endpoint as a substitute. A difference in the post-transplant transaminase levels or incidence of delayed graft function have often been used, or markers proving less ischaemia-reperfusion injury which has been a reported endpoint with many MP series [11•, 15•, 17•, 28•, 40, 49]. The circulating perfusate in the liver itself prevents accumulation of succinate and other metabolic products and removes the debris and necrotic or apoptotic cells, likely decreasing the post-reperfusion transaminases and having a beneficial effect on the graft [19•, 50]. The major benefit is, however, the oxygenation preventing the damage caused by ischaemia and anaerobic metabolism [50]. The pre-clinical research gathered mechanistic evidence of multiple aspects of cellular metabolism pathways influenced by machine perfusion with several teams focused in particular on energy metabolism and mitochondria function [51]. The HOPE perfusion was shown to induce mitochondrial function with down-regulation of the respiratory rate, associated with adenosine tri-phosphate synthesis. Such 60 to 120-min duration perfusion achieves recovery of the liver energy resources together with prevention of mitochondrial reversal flow of electrons during the organ re-warming, decreasing production of reactive oxygen species mitigating activation of the inflammatory processes involved in the reperfusion injury [52, 53].

Despite an extensive knowledge on a cellular level of the mechanisms of hepatic function protection during HOPE, NMP-L has the advantage of allowing the exploration of targeted therapeutic interventions analogous to conventional medical approaches. For example, to combat the anticipated risks of bacterial contamination and overgrowth, antibiotics are universally added to the normothermic perfusate fluid composition [30, 32]. Targeted antibiotics added to the perfusate for livers from positive culture donors might be an easy intervention to improve transplantability of organs from donors with infections. A proof of concept with NMP-L antiviral pre-treatment during machine perfusion in a porcine model was recently reported by the Toronto group [54]. The same group also explored strategies to further enhance the protective MP mechanisms in a sub-normothermic porcine DCD liver model, enriching the perfusate with anti-inflammatory drugs (alprostadil, n-acetylcysteine, carbon monoxide and sevoflurane). This intervention significantly lowered the perfusate levels of aspartate aminotransferase, interleukin 6, tumour necrosis factor alpha and galactosidase, and increased interleukin 10 levels compared to the untreated controls. Machine perfusion itself reduces activation of the post-reperfusion inflammation cascade, and despite the improvements in reperfusion injury markers not achieving statistical significance, this concept to enhance the protective mechanisms merits further research [52, 53].

Another frequent reason for discarding donor livers is a suboptimal flushing. Addition of a thrombolytic agent together with the standardly included heparin might improve graft circulation without increasing the bleeding risks for the organ recipient [31, 55].

Steatotic livers are the largest group of poorly utilised organs, and resuscitation of fatty livers is an important goal whilst combating the growing obesity epidemic [56]. The concept of pharmacological intervention to reverse steatosis during NMP-L has been explored by several teams including our own. We performed NMP-L on severely steatotic donor human livers, exposing the organs to a combination of de-fatting drugs. We observed solubilisation of the liver fat, commencing within 3 h, and continuing until the end of 24-h perfusions (unpublished data). Although it is unclear whether removal of fat from a viable liver during NMP-L would be relevant in improving its post-transplant function and outcome, the treated organs showed significantly better metabolic parameters compared to matched controls, and the histological improvement became apparent after only 6 h. A potential shortfall of machine perfusion with metabolically active livers might be re-circulation of harmful or toxic metabolites [57]. In the described experiment the mitochondria fatty acids β-oxidation increased ketone production and continuously increased the perfusate apolipoprotein and cholesterol levels. Removal of the metabolism by-products from the device circuit is an interesting issue requiring more research.

The perfusion model of steatotic livers also demonstrates that different graft categories may benefit from different machine perfusion modalities. Cooling the hepatic fat leads to changed lipid consistency, increasing its droplet volume which ultimately compromises the liver microcirculation. The optimal strategy to minimise any post-procurement damage might be minimising exposure to the cold by normothermic preservation. Following a period of SCS, however, the disturbed hepatic microcirculation makes subsequent machine resuscitation and perfusion processes challenging and the optimal temperature and combination of different approaches is yet to be determined.

The feasibility of the therapeutic interventions discussed has been demonstrated by animal or proof of concept human research. In the near future, MP will be studied as a method to deliver cell-based and small molecule therapies, aimed at improving the condition of high-risk liver grafts, following the emerging evidence showing efficacy of these novel concepts in promoting organ regeneration [58–61].

## Conclusion

Machine perfusion is a rapidly progressing field which is likely to change multiple aspects of liver preservation and transplantation practice in the future. This superior organ preservation mode has already shown benefits by enabling functional liver assessment with normothermic perfusion or reduction of non-anastomotic biliary strictures by hypothermic perfusion. Machine perfusion looks likely to set new limits for organ preservation times, increase utilisation of the highest-risk DBD grafts and improve long-terms outcomes in DCD livers. The perfusion procedure will provide an opportunity for liver regeneration and therapeutic intervention. Different type of livers may benefit from different perfusion strategies or their combination.

## Abbreviations

ALT: Alanine transaminase
CIT: Cold ischaemic time
COPE: Consortium for Organ Preservation in Europe
DBD: Donation after brain death
DCD: Donation after circulatory death
ECD: Extended criteria donors
HBOC: Haemoglobin-based oxygen carrier
HMP-L: Hypothermic machine perfusion of the liver
HOPE: Hypothermic oxygenated perfusion
MP: Machine perfusion
NMP-L: Normothermic machine perfusion of the liver
RBC: Red blood cells
SCS: Static cold storage
